# Spatiotemporal regulation of the bone immune microenvironment via a ‘Zn^2+^-quercetin’ hierarchical delivery system for bone regeneration

**DOI:** 10.1093/rb/rbaf006

**Published:** 2025-02-13

**Authors:** Hengliang Sun, Yedan Chen, Xiaoqin Sang, Qingxiang Liu, Haoran Yu, Shaojun Hu, Yingji Mao, Li Zhang

**Affiliations:** Graduate School, Anhui Medical University, Hefei, Anhui 230032, China; Department of Plastic Surgery, The First Affiliated Hospital of Bengbu Medical University, Bengbu, Anhui 233004, China; Department of Plastic Surgery, Second People’s Hospital of Wuhu City, Wuhu, Anhui 241001, China; Department of Plastic Surgery, The First Affiliated Hospital of Bengbu Medical University, Bengbu, Anhui 233004, China; Anhui Nerve Regeneration Technology and Medical New Materials Engineering Research Center, School of Life Sciences, Bengbu Medical University, Bengbu, Anhui 233030, China; Anhui Nerve Regeneration Technology and Medical New Materials Engineering Research Center, School of Life Sciences, Bengbu Medical University, Bengbu, Anhui 233030, China; Department of Rehabilitation Medicine, The First Affiliated Hospital of Bengbu Medical University, Bengbu, Anhui 233004, China; Anhui Nerve Regeneration Technology and Medical New Materials Engineering Research Center, School of Life Sciences, Bengbu Medical University, Bengbu, Anhui 233030, China; Department of Rehabilitation Medicine, The First Affiliated Hospital of Bengbu Medical University, Bengbu, Anhui 233004, China; Anhui Nerve Regeneration Technology and Medical New Materials Engineering Research Center, School of Life Sciences, Bengbu Medical University, Bengbu, Anhui 233030, China; Department of Orthopedics, Huaiyuan County People’s Hospital, Bengbu, Anhui 233400, China; Department of Plastic Surgery, The First Affiliated Hospital of Bengbu Medical University, Bengbu, Anhui 233004, China; Anhui Nerve Regeneration Technology and Medical New Materials Engineering Research Center, School of Life Sciences, Bengbu Medical University, Bengbu, Anhui 233030, China; Anhui Provincial Key Laboratory of Tumor Evolution and Intelligent Diagnosis and Treatment, Bengbu Medical University, Bengbu, Anhui 233030, China; Graduate School, Anhui Medical University, Hefei, Anhui 230032, China; Department of Plastic Surgery, The First Affiliated Hospital of Bengbu Medical University, Bengbu, Anhui 233004, China

**Keywords:** metal-organic framework, quercetin, bone immune regulation, hierarchical delivery, bone defect

## Abstract

The immunoregulation of tissue-engineered bone has emerged as a prominent area for bone defect repair. While this field demonstrates considerable potential, effectively managing relevant factors and maintaining a balanced immune microenvironment in practical applications remain substantial challenges that require resolution. In this study, we tested a novel comprehensive hierarchical delivery system based on the requirements of a natural immune microenvironment for inflammatory factors, to optimize local immune responses through precise regulation of drug release. Quercetin (Que)-loaded zeolite imidazolate framework-8 (ZIF-8) nanoparticles were embedded in gelatin methacrylate to create a drug-release system featuring a Zn^2+^ shell and quercetin core. *In vivo* and *in vitro* studies demonstrated that this dual sustained-release hydrogel-ZIF-8 system can produce low concentrations of Zn^2+^ at an early stage, resulting in a mild anti-inflammatory effect and proliferation of bone marrow mesenchymal stem cells. Moreover, as inflammation advances, the release of quercetin works synergistically with Zn^2+^ to enhance anti-inflammatory responses, reconfigure the local microenvironment, and mitigate the inflammatory response that adversely impacts bone health by inhibiting the Nuclear Factor-kappa B (NF-κB) signaling pathway, thereby promoting osteogenic differentiation. This system is pioneering for sequential microenvironment regulation based on its diverse anti-inflammatory properties, offering a novel and comprehensive strategy for bone immune regulation in the clinical treatment of bone defects.

## Graphical abstract

**Figure:**
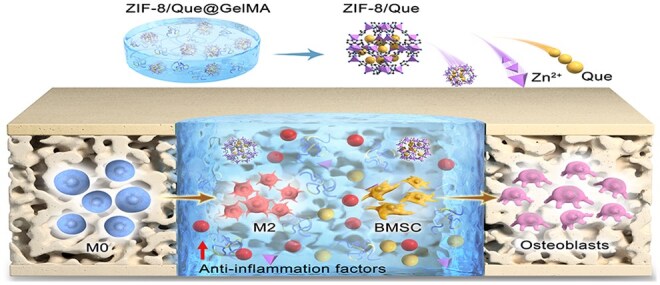

## Introduction

Clinicians continue to face challenges in repairing bone defects. Although minor bone defects typically heal independently [1], larger and more severe defects can require external intervention to facilitate bone regeneration and ensure overall health [2]. Autologous bone grafts remain the gold standard for treating large bone defects [3]. However, they are associated with severe drawbacks, including donor site morbidity and graft size restrictions [4]. Therefore, leveraging technologies such as stem cells and biomaterials to construct scaffolds that mimic the structure of natural bone in tissue engineering provides an innovative approach to repairing bone defects [5, 6].

The relationship between immune regulation and bone regeneration, especially the differentiation of bone marrow mesenchymal stem cells (BMSCs), is complex and has gained significant attention in recent research [7, 8]. BMSCs play a crucial role in the bone healing process and can differentiate into osteoblasts, which are essential for bone formation. However, their differentiation and functionality are significantly influenced by the surrounding immune environment. A key factor in this relationship is how immune cells, particularly macrophages, modulate the behavior of BMSCs. Macrophages can assume various phenotypes, predominantly M1 (pro-inflammatory) and M2 (anti-inflammatory), which profoundly influence the bone regeneration process. M1 macrophages release inflammatory signals like Tumor Necrosis Factor-alpha (TNF-α) and Interleukin-1 beta (IL-1β) [9, 10], which can impede the ability of BMSCs to differentiate into bone-forming cells. Conversely, M2 macrophages encourage the secretion of anti-inflammatory cytokines via paracrine signaling, fostering the establishment of a healing environment and steering the polarization of BMSCs toward the osteogenic lineage [9]. Among these, IL-10 has been recognized as a crucial factor that not only diminishes inflammation but also enhances the osteogenic differentiation of BMSCs [11]. Consequently, a more profound understanding of the interactions between immune cells and BMSCs not only offers new perspectives on the mechanisms of bone healing but also establishes a significant foundation for the advancement of innovative therapeutic strategies.

The inflammatory microenvironment plays a dual role in the process of bone healing; while moderate inflammation supports the healing process, excessive inflammation can cause delays or prevent healing [12]. The NF-κB signaling pathway is a key regulator of the inflammatory response. Its importance in bone healing is receiving increasing attention. Studies indicate that NF-κB activation is closely linked to the survival, proliferation and differentiation of bone cells [13]. Quercetin, a component of the Chinese medicine *Sambucus williamsii* [14], has minimal side effects [15] and notable anti-inflammatory properties [16, 17], which may positively impact bone healing by regulating NF-κB activity. Our previous research involved loading quercetin into solid lipid scaffolds for sustained release, promoting M2 macrophage polarization [8]. Current research on immunomodulatory treatments for bone healing primarily emphasizes maintaining strong anti-inflammatory effects [18, 19]. However, an early diminished anti-inflammatory impact is equally crucial for successful outcomes. This can activate appropriate levels of pro-inflammatory macrophages and factors, facilitating the recruitment of mesenchymal stem cells, osteogenic progenitor cells and vascular progenitor cells to bone defects, thereby promoting osteogenesis [20]. Therefore, we aimed to develop a sustained-release system offering low anti-inflammatory effects while capturing quercetin. This quercetin is subsequently released to produce strong anti-inflammatory effects, enabling spatiotemporal regulation of the immune response.

Zeolite imidazole framework-8 (ZIF-8), a significant subclass of metal-organic frameworks, has a large specific surface area, high porosity and stable degradation characteristics [21]. Its porous design allows ZIF-8 to achieve high drug-loading efficiency, which is essential for developing drug-delivery systems [22]. In addition, ZIF-8 is sensitive to pH changes [23], allowing it to release Zn^2+^ responsively and exhibit anti-inflammatory effects [24]. ZIF-8 nanoparticles loaded with angiogenic drugs can sequentially release Zn^2+^ and angiogenic drugs as they decompose, aligning this release with the various stages of wound healing [25]. However, these bioactive nanoparticles have limited immunomodulatory properties, which can be improved by loading quercetin [26]. Moreover, Zn^2+^ can be toxic and cause adverse reactions at high concentrations, so it is crucial to maintain a safe release concentration [27]. GelMA hydrogels have a unique microporous structure and good biodegradability, making them excellent carriers that can be easily shaped for minimally invasive injections [28, 29]. Therefore, a dual-release system that combines GelMA hydrogels with ZIF-8 would effectively stabilize the release of zinc ions at lower concentrations, ensuring their safety and effectiveness.

**Figure 1.**
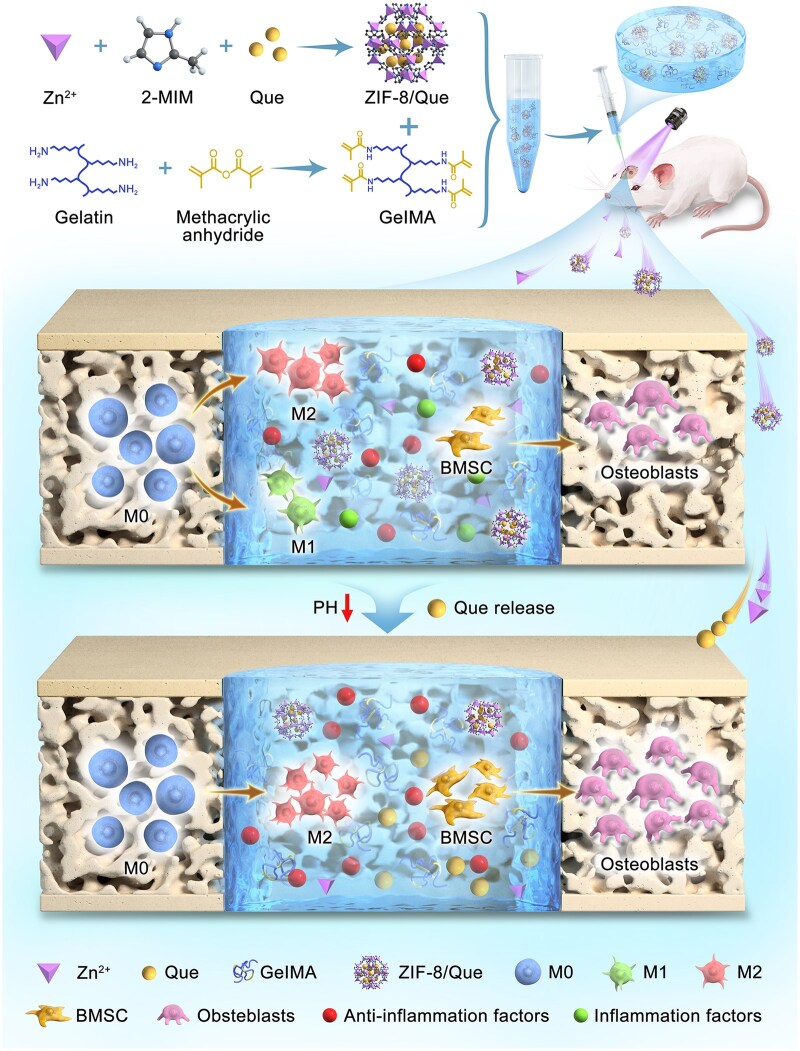
Schematic illustration of a hierarchical ZIF-8/Que@GelMA delivery system for sequential Zn^2+^ and quercetin (que) to promote bone defect repair by regulating the immune microenvironment.

This study aimed to develop an injectable hierarchical drug-delivery system by integrating ZIF-8/quercetin (ZIF-8/Que) nanoparticles with GelMA hydrogel (ZIF-8/Que@GelMA) for immune modulation and bone regeneration in bone defect repair (Figure 1A). The system enables the sequential release of Zn^2+^ and quercetin, targeting different stages of bone immune response to achieve dual effects of anti-inflammation and bone differentiation. We designed a series of *in vitro* and *in vivo* experiments to evaluate its potential in bone tissue repair, focusing on the system’s ability to modulate the immune microenvironment and its application in bone defect healing. Through these experiments, we aimed to assess the system's biocompatibility, anti-inflammatory properties and bone regeneration efficacy, offering new strategies for bone tissue engineering and immunotherapy.

## Materials and methods

### Materials

Zinc nitrate hexahydrate [Zn(NO_3_)_2_·6H_2_O] and 2-methylimidazole (2-MIM) were purchased from Shanghai Aladdin Co. (Shanghai, China). Quercetin was purchased from Shanghai Macklin Biochemical Co. (Shanghai, China).

### ZIF-8 and ZIF-8/Que nanoparticle synthesis

Quercetin was embedded into ZIF-8 via one-step synthesis. Previous studies used a quercetin dosage between 5 and 10 mg, and we tested 8 mg for the experiment while ensuring the stability of the nanoparticle shape [24, 30]. Specifically, 2-MIM (330 mg) was dissolved in 10 mL of methanol, and 150 mg of Zn(NO_3_)_2_·6H_2_O was dissolved in 5 mL of methanol. These two solutions were mixed, and 8 mg of quercetin was immediately added. The reaction mixture was vigorously stirred for 30 min to obtain a yellow emulsion-like suspension. Finally, yellow ZIF-8/Que nanoparticles were obtained via centrifugation at 13 000 rpm and were washed three times with methanol; the supernatant was collected for drug-loading efficiency (DLE) and drug encapsulation efficiency (DEE) detection. Pure ZIF-8 was synthesized similarly, using 330 mg of 2-MIM (dissolved in 10 mL of methanol) and 150 mg of Zn(NO_3_)_2_·6H_2_O (dissolved in 5 mL of methanol), mixed without the addition of quercetin.

### ZIF-8 and ZIF-8/Que nanoparticle characterization

The morphology and particle size of the nanoparticles were examined using transmission electron microscopy (TEM, JEOL JEM-F200, Japan) and scanning electron microscopy (SEM, TESCAN MIRA LMS, Czech Republic) to characterize the samples. Energy-dispersive X-ray spectrometry (EDS) was used with TEM to identify the presence of zinc (Zn). X-ray diffraction (XRD) patterns were acquired using a Rigaku X-ray diffractometer (Rigaku, Japan). The functional groups were investigated through Fourier transform infrared (FT-IR) spectrometry (Thermo, Nicolet 6700, USA), covering a wavelength range of 400–4000 cm^−1^. To determine the DLE and DEE, an ultraviolet-visible (UV-vis) absorption spectrum (Thermo, USA) was used to establish a standard curve for optical density measurements at a wavelength of 370 nm (Y = 0.0772X − 0.052; *R*^2^ = 0.9989).

### ZIF-8/Que@GelMA scaffold preparation

The GelMA hydrogels were synthesized using a method we previously established [28]. Briefly, methacrylate anhydride (EFL, China) was gradually dissolved in a gelatin solution consisting of gelatin and preheated phosphate-buffered saline (PBS; Gibco, China). After a complete reaction lasting 2 h, unbound impurities were removed using a dialysis bag (Molecular Weight: 8000–14 000). After 7 days of continuous dialysis, the dialysate was heated to 60°C and filtered using a 0.22-μm filter membrane. Finally, freeze-drying produced a porous white foam ready for use.

ZIF-8/Que@GelMA was prepared by combining ZIF-8/Que nanoparticles with lyophilized GelMA. A total of 25 mg of the photoinitiator LAP was dissolved in 10 mL of PBS at 50°C until complete dissolution occurred. GelMA (0.5 g) was added to this mixture. We chose a 5% GelMA concentration for the experiment. This concentration is advantageous because it creates a controlled and sustained-release curve [31], resulting in optimal dual-release effects, whereas higher concentrations adversely affect cell diffusion and proliferation [32]. The optimal concentration was then evaluated by examining the impact of varying concentrations (10, 25, 50, 100, 200 and 400 μg/mL) of ZIF-8/Que on cell proliferation. The ZIF-8/Que (100 μg/mL) was then dispersed in the GelMA solution and stirred overnight at 40°C in a dark environment. Finally, the solution was photocured in gel under 405 nm UV light and freeze-dried to produce ZIF-8/Que@GelMA. ZIF-8@GelMA was prepared using the same procedure.

### ZIF-8/Que@GelMA scaffold characterization

To examine the structure of the hydrogel scaffold, it was first lyophilized and then transversely sectioned to prepare the samples. After gold plating, SEM and EDS were employed to analyze the morphology and elemental composition. The samples were positioned on a contact-angle test platform (Kruss, Germany) to evaluate the hydrophilicity of the hydrogel scaffolds. A drop of deionized water (10 μl) was automatically dispensed onto the sample surface, and photographs were taken during the test to calculate the water contact angle.

To measure the swelling rate of the hydrogel scaffolds, we dried and weighed each sample (denoted as W0). Then, samples were placed into distilled water, and excess water on the surface of the hydrogel was absorbed using filter paper at various time intervals. The swelling mass of the hydrogels (W1) was recorded until equilibrium was reached. The swelling rate was calculated using the following formula: swelling rate (%) = (W1 − W0)/W0 × 100%.

To investigate the mechanical properties, samples were prepared as cylinders with heights and diameters of 8 mm. The average fracture stress was determined using an electronic universal testing machine (Instron, USA) operating at a constant speed of 0.5 mm/min until the samples were fractured. Subsequently, the pressure–strain curve was plotted based on the results, and Young’s modulus was calculated.

To simulate the release of Zn^2+^ and quercetin under acidic conditions in an inflammatory environment and neutral conditions in a physiological environment, ZIF-8/Que@GelMA was immersed in 2 mL of a culture medium at pH 6.0 and 7.4. Samples were collected at 1 h, 3 h, 6 h, 12 h, 1 day, 2 days, 3 days, 5 days, 7 days, 10 days, 14 days, 30 days, 45 days and 60 days. The leachate was analyzed using inductively coupled plasma optical emission spectrometry (Agilent 5110, USA) to determine the concentration of Zn^2+^ in the samples. The concentration of quercetin was quantified based on UV-vis absorption, and its absorbance was measured at 370 nm.

### BMSC biocompatibility on different scaffolds

For *in vitro* studies, rat BMSCs were harvested and maintained according to the procedures described in our previous studies [33, 34]. Cells sustained at 3–4 passages were used in all experiments. BMSCs were planted on hydrogels to assess cell viability on days 1, 2 and 3; a Calcein-AM/PI Live/Dead Assay Kit (Beyotime) was used to label both live and dead cells. After staining, the findings were documented using an inverted fluorescence microscope (Zeiss Axio Observer Z1, Germany). Concurrently, a Cell Counting Kit-8 (CCK-8, Beyotime, China) was used to evaluate the proliferation rates and adhesion ability of BMSCs in various groups, with the optical density measured at 450 nm.

BMSCs were plated in a 24-well plate and incubated for 3 days to examine cell morphology following adhesion to the hydrogel scaffold. After fixation with 4% paraformaldehyde for 30 min, the samples were treated with fluorescein isothiocyanate (FITC)-phalloidin (Solarbio, China) in the dark for 1 h and counterstained with 4',6-diamidino-2-phenylindole (DAPI) (Biosharp, China) for 15 min. The influence of the hydrogel on the migration capability of BMSCs was evaluated using scratch-healing assays. BMSCs were introduced into 6-well plates and cultured until 90% confluence was achieved. Following treatment with various materials, a scratch was made using the end of a 200-μl sterile pipette, and cells were cultured for 12 and 24 h. After 30 min of staining with Calcein-AM (Beyotime, China), fluorescent images were captured, and the extent of wound closure was quantified using ImageJ software (NIH, USA).

### Osteogenic differentiation of BMSCs *in vitro*

#### Alkaline phosphatase activity

Alkaline phosphatase (ALP) activity serves as a standard biochemical marker for osteoblasts and was used to assess the osteogenic differentiation of BMSCs. BMSCs (1 × 10^4^ cells/well) were seeded on the hydrogels in an osteogenic induction medium (Oricell, New Zealand). ALP staining was conducted on day 7 using an ALP staining kit (Beyotime, China), and images were captured using an inverted microscope. Subsequently, the supernatant was extracted and analyzed for ALP activity according to the manufacturer’s protocol.

#### Alizarin red staining

BMSCs (1 × 10^4^ cells/well) were cultivated on the hydrogel in a 24-well plate to examine the mineralized osteoblast nodules. After culturing for 21 days, the samples were preserved in 4% paraformaldehyde for 15 min and subsequently stained with 2% alizarin red (Solarbio, China) for 30 min. Calcium nodule formation was assessed using an inverted microscope.

#### Immunofluorescence staining

The expression level of Runx2 in BMSCs was evaluated via immunofluorescence after 7 days of culture, whereas Osteocalcin (OCN) expression was assessed after 14 days. BMSCs were fixed with 4% paraformaldehyde, permeabilized with 0.1% Triton X-100 (Solarbio) and blocked with 5% BSA buffer (Solarbio) at room temperature. The cells were incubated overnight at 4°C with antibodies specific to Runx2 (1:400, Servicebio, China) or OCN (1:100, Affinity). The cells were then treated with fluorescent secondary antibodies (1:100, affinity) for 1 h. Nuclear staining was performed using DAPI. Finally, the cells were analyzed using a fluorescence microscope, and the relative fluorescence intensity was quantified using the ImageJ software.

### Effects in the bone immune environment *in vitro*

#### Polarization of macrophages

To simulate the bone immune microenvironment, bone marrow-derived macrophages (BMDMs) were harvested from male Sprague–Dawley (SD) rats aged 4 weeks, following the extraction procedures outlined in our previous study [28]. BMDMs, at a cell density of 2 × 10^5^ cells/mL, were cultured in a medium supplemented with 20-ng/mL macrophage colony-stimulating factor for 4 days. Subsequently, the cells were incubated with the hydrogel and 200-ng/mL lipopolysaccharide (LPS) in a fresh culture medium. After 3 days, the cells were stained with FITC-labeled anti-CD86 and APC-labeled anti-CD206 antibodies. Images were captured using a fluorescence microscope. Similarly, PBS-treated cells were washed and incubated on ice in the dark with fluorescently labeled flow antibodies. After 30 min, the cells were rewashed with PBS and resuspended, and then the fluorescence intensity of the target marker was assessed using flow cytometry.

#### Enzyme-linked immunosorbent assay

To measure inflammatory factors, BMSCs were first prepared at a density of 5 × 10^4^ cells/mL. Subsequently, 1 mL of the BMSC suspension was seeded into each well of a 24-well plate that already contained hydrogel. After the cells adhered, the medium was replaced with one containing 200 ng/mL of LPS to initiate the simulation of the inflammatory microenvironment. After incubating for 1 and 3 days, the expression levels of IL-1β, TNF-α, and IL-10 in the supernatants were measured using an enzyme-linked immunosorbent assay (ELISA) kit (Elabscience, China).

#### Western blot assay

After cultivating BMSCs on the composite hydrogel surface for 10 days, cells were lysed on ice with RIPA cell lysis buffer (EpiZyme, China) to extract proteins. The protein concentration in the supernatant was then assessed using a BCA kit. The proteins were subsequently transferred to a polyvinylidene fluoride membrane (Beyotime; China) via sodium dodecyl sulfate-polyacrylamide gel electrophoresis (EpiZyme; China). The membrane was blocked in a blocking solution (Beyotime; China) for 15 min and incubated overnight with the following primary antibodies: β-tubulin (1:5000, Proteintech), β actin (1:5000, Affinity), IL-10 (1:2000, Proteintech), Runx2 (1:500, Affinity), NF-κB p65 (1:500, Affinity) and p-NF-κB p65 (1:500, Affinity). After another round of washing, the membrane was incubated with the secondary antibody at room temperature for 1 h. The immunoreactive bands were visualized using a multifunctional imaging system SH-compact523 (Shenhua Technology Co., China) and quantified for relative band intensity using ImageJ.

### *In vivo* animal studies

#### Establishment of cranial defect model in rats

All animal procedures and treatments were approved by the Ethics Committee of the Bengbu Medical University (Approval No. 2021272). The 6-week-old male SD rats were procured from the Qinglongshan Experimental Animal Research Center (Nanjing, China). The rats were divided into GelMA, ZIF-8@GelMA, ZIF-8/Que@GelMA, and control groups (without any materials). Following intraperitoneal injection of 2% pentobarbital for anesthesia and skin preparation, an electric drill created a 5-mm diameter critical-size bone defect on both sides of the calvarium. The hydrogel was injected to fill the defect sites and cured under UV light irradiation. The periosteum and skin were then closed and disinfected. Rats were euthanized at 4 or 8 weeks postoperatively, and the harvested craniums were fixed in 4% paraformaldehyde for histological analysis.

#### Micro-CT analysis

Rat cranial specimens were collected 8 weeks post-surgery, and imaging analysis was conducted using a micro-CT imaging system (GE, USA) to assess the progress of bone repair. The defect site was analyzed to calculate the percentage of new bone volume relative to the total volume (BV/TV), as well as bone mineral density (BMD), trabecular number (Tb. N) and trabecular thickness (Tb. Th).

#### Histological analysis

All cranium specimens were decalcified, embedded in paraffin and sectioned into 5-μm-thick slices using a microtome (Wetzlar, Germany). Hematoxylin & eosin (H&E) and Masson’s trichrome staining were performed, and samples were examined microscopically to evaluate bone formation. Additionally, sections were incubated overnight at 4°C with primary antibodies against IL-1β (1:100, Affinity), TNF-α (1:100, Affinity), IL-10 (1:100, Affinity), CD86 (1:100, Affinity), CD206 (1:100, Affinity), Collagen Type I (COL-1) (1:100, Affinity), OCN (1:1000, Proteintech) and Runx2 (1:1000, Proteintech). For IL-1β, TNF-α and IL-10, sections were subsequently incubated with a horseradish peroxidase-conjugated secondary antibody (1:200, Affinity). Nuclei were counterstained with diaminobenzidine and hematoxylin, and stained tissue images were captured using a digital pathology scanner (3D Histech, Hungary). The remaining sections were stained with Cy3-labeled (1:1000, Proteintech) or FITC-labeled (1:1000, Proteintech) secondary antibodies for 2 h at room temperature and counterstained with DAPI for 10 min, and images were acquired under a fluorescence microscope.

### Statistical analysis

The data analyzed in this study were processed using the GraphPad Prism 8 software (GraphPad Software, USA) and are presented as the mean ± standard error. Statistical significance was assessed using one-way analysis of variance and Student’s *t*-test at a significance level of *P *<* *0.05. Data are indicated as *P <* *0.05, **P <* *0.01, and ***P <* *0.001; “ns” indicates no statistical significance.

## Results

### ZIF-8/Que nanoparticle preparation and characterization

**Figure 2.**
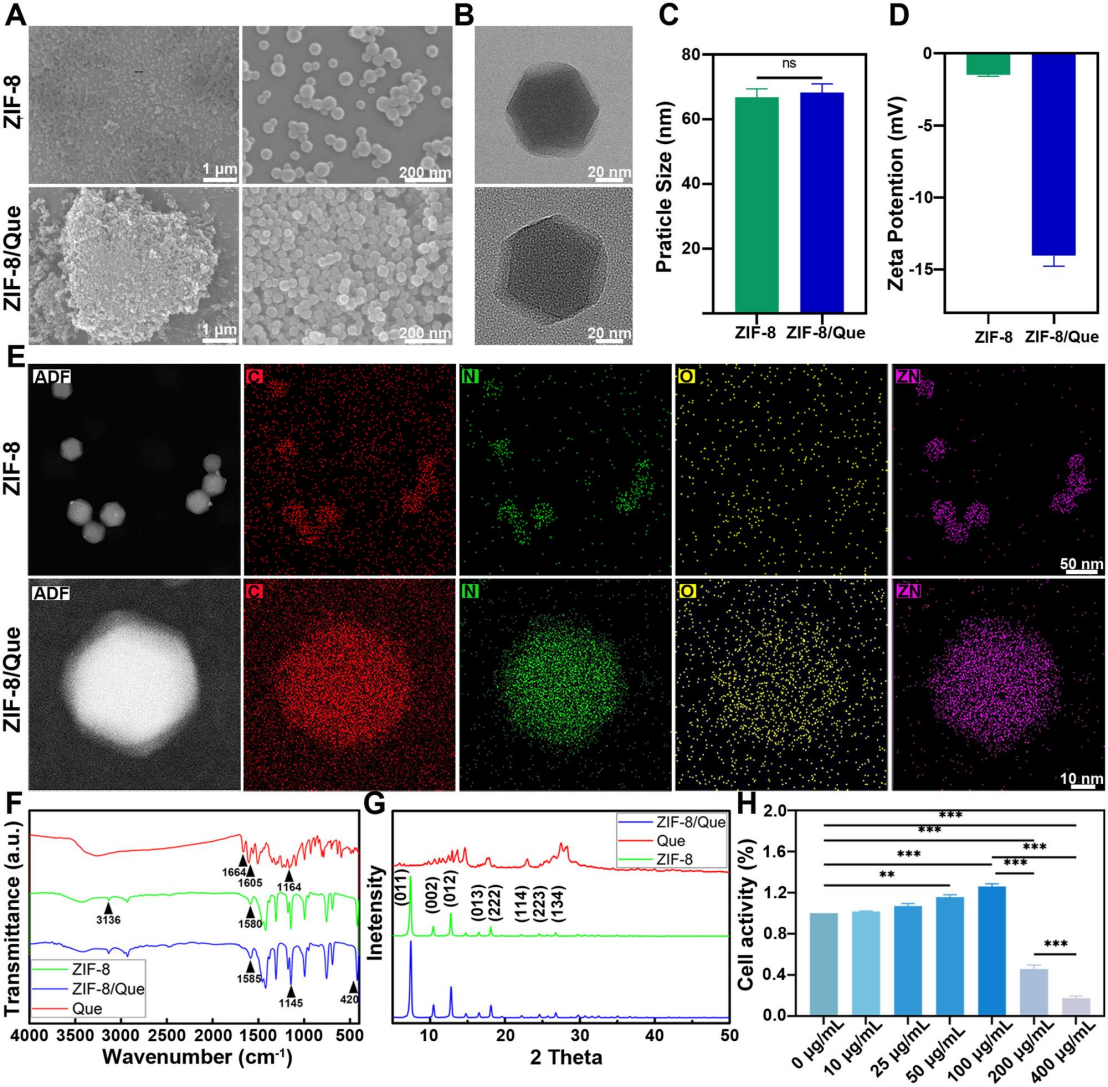
Morphology and characterization of nanoparticles. (**A**) SEM images, (**B**) TEM images, (**C**) particle size, (**D**) Zeta potential, (**E**) EDS images, (**F**) FT-IR spectra and (**G**) XRD of que sample, ZIF-8 and ZIF-8/que. (**H**) Cell activity of various concentrations of ZIF-8/que on BMSCs (*n* = 3, *P <* *0.05, **P <* *0.01 and ***P <* *0.001, ns: not significant).

ZIF-8/Que nanoparticles were synthesized using a one-pot method to load quercetin into ZIF-8 [31]. The SEM and TEM images revealed that both the ZIF-8 and ZIF-8/Que nanoparticles exhibited similar dodecahedral morphologies, with particle sizes ranging from 80 to 100 nm (Figure 2A–C). Owing to the presence of many phenolic hydroxyl groups on the surface of quercetin, the negative value of the zeta potential increased after its loading, which is consistent with previous research [35] (Figure 2D). Furthermore, the presence of oxygen (O), which is absent in ZIF-8 (Supplementary Figure S1A), in the EDS spectrum of ZIF-8/Que confirmed the successful incorporation of quercetin (Figure 2E).

In further verifying the successful fabrication of ZIF-8/Que, the FT-IR spectra revealed that ZIF-8 and ZIF-8/Que exhibited absorption peaks at 3136, 1145 and 420 cm^−1^, which are consistent with the findings from previous studies [36]. This characteristic absorption corresponded to the vibrational modes of the C–H bond, C–N bond and Zn–N functional groups of the methyl group. In the quercetin spectrum, the C=O absorption peak at 1664 cm^−1^ and C–CO–C ketone stretching and bending at 1164 cm^−1^ were absent in the ZIF-8/Que spectrum. Only the enhanced formant corresponding to the C=N bond in the imidazole ring of ZIF-8 at 1580 cm^−1^ and benzene ring of quercetin at 1605 cm^−1^ appeared as a combined peak at 1585 cm^−1^ in the ZIF-8/Que composite. This suggests that a significant portion of quercetin was encapsulated within ZIF-8, resulting in a shielding effect (Figure 2F). Furthermore, XRD pattern analysis revealed that ZIF-8 and ZIF-8/Que exhibited similar crystal characteristic diffraction peaks, indicating that the incorporation of quercetin did not alter the crystal structure of ZIF-8 (Figure 2G).

As measured via UV spectrophotometry, the DLE and DEE of ZIF-8/Que were 21.26% and 90.9%, respectively. High concentrations of ZIF-8 are toxic, with previous studies indicating that concentrations ranging from 75 to 100 μg/mL inhibit cell proliferation [37]. This study employed the CCK-8 method to evaluate the tolerable concentration of ZIF-8/Que nanoparticles affecting BMSCs’ survival rate. The findings indicated that when the concentration surpassed 100 μg/mL, cell growth was significantly inhibited. Consequently, a concentration of 100 μg/mL of ZIF-8/Que was selected for further investigation (Figure 2H).

### ZIF-8/Que@GelMA characterization

**Figure 3.**
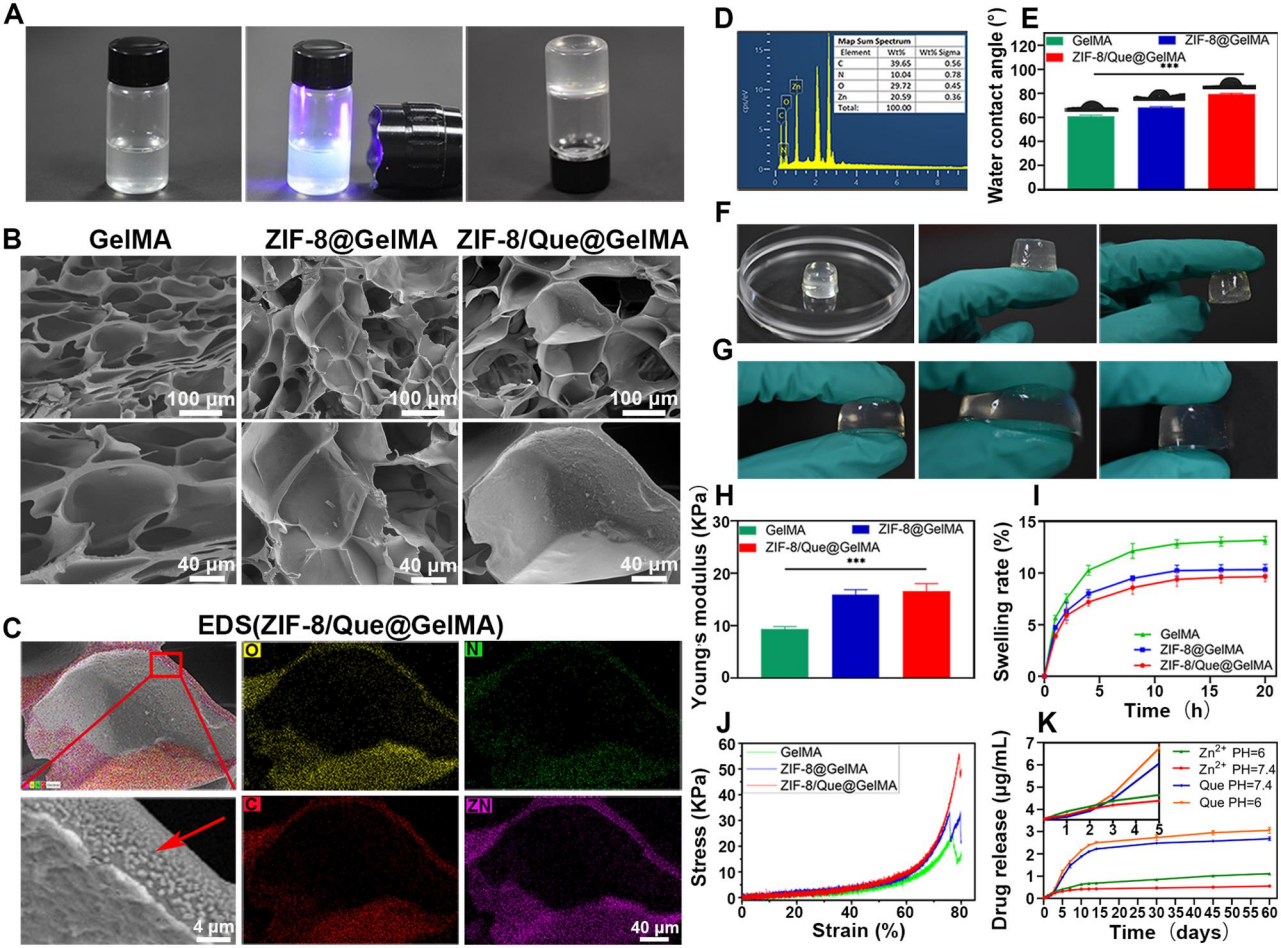
Morphology and characterization of the hydrogel. (**A**) Photographs of the gelation process, (**B**) SEM images. EDS images (**C**) and elemental composition (**D**) of ZIF-8/Que@GelMA. (**E**) Water contact angle. Adhesion properties (**F**) and elasticity (**G**) of ZIF-8/Que@GelMA. Young’s modulus (**H**), swelling ratio (**I**) and pressure–strain curve (**J**) of hydrogel scaffolds. (**K**) Drug-release curve of Zn^2+^ and que in pH 6.0 and pH 7.4 (*n* = 3, *P <* *0.05, **P <* *0.01 and ***P <* *0.001).

Through crosslinking via UV irradiation, GelMA hydrogels could be fabricated into various shapes (Figure 3A and Supplementary Figure S1C); this is advantageous for repairing complex-shaped bone defects. As illustrated in Figure 3B, the cross-section of the GelMA hydrogel exhibited a porous sponge-like structure. The ZIF-8@GelMA and ZIF-8/Que@GelMA hydrogels also had large pores, and the cross-section revealed rough and convex particles (Supplementary Figure S1B), corresponding to the presence of ZIF-8 nanoparticles, confirmed via EDS (Figure 3C and D). Compared with the GelMA alone, the water contact angle of ZIF-8/Que@GelMA increased, indicating a decrease in hydrophilicity. This change contributed to maintaining cleaner surfaces (Figure 3E). However, the adhesion ability remained unaffected, possibly because of the increased hydrogel roughness (Figure 3F).

The GelMA hydrogel was also characterized by low mechanical strength. Incorporating metal-frame nanoparticles enabled the ZIF-8/Que@GelMA to retain good elasticity while enhancing its mechanical strength (Figure 3G). Compared with those of GelMA, ZIF-8/Que@GelMA exhibited a higher Young’s modulus and lower swelling rate, which would help the material to maintain a proper fit with the surrounding tissue (Figure 3H and I), thereby minimizing nerve compression in that area. The pressure–strain curve demonstrated that the maximum pressure value at the breaking point of ZIF-8/Que@GelMA was nearly twice that of GelMA, highlighting its superior performance in resisting damage from external forces and making it an ideal candidate for osteogenic scaffold applications (Figure 3J).

The Zn^2+^ and quercetin release curves demonstrated hierarchical release characteristics. Initially, Zn^2+^ exhibited low-concentration release owing to the sustained-release properties of the hydrogel, whereas quercetin was almost entirely retained as it was encapsulated within ZIF-8. As the concentration of Zn^2+^ increased, the metal framework of ZIF-8/Que@GelMA began to dissolve, leading to the significant release of quercetin. Subsequently, the material was placed in an acidic solution (pH 6) to simulate the microenvironment of bone defects following increased inflammation. In this acidic environment, Zn^2+^ and quercetin were released more rapidly from the ZIF-8/Que nanoparticles than in a neutral environment (Figure 3K). These results demonstrated the ability to achieve hierarchical, sustained and pH-responsive drug release, effectively targeting acidic environments.

### Hierarchical delivery system biocompatibility

**Figure 4.**
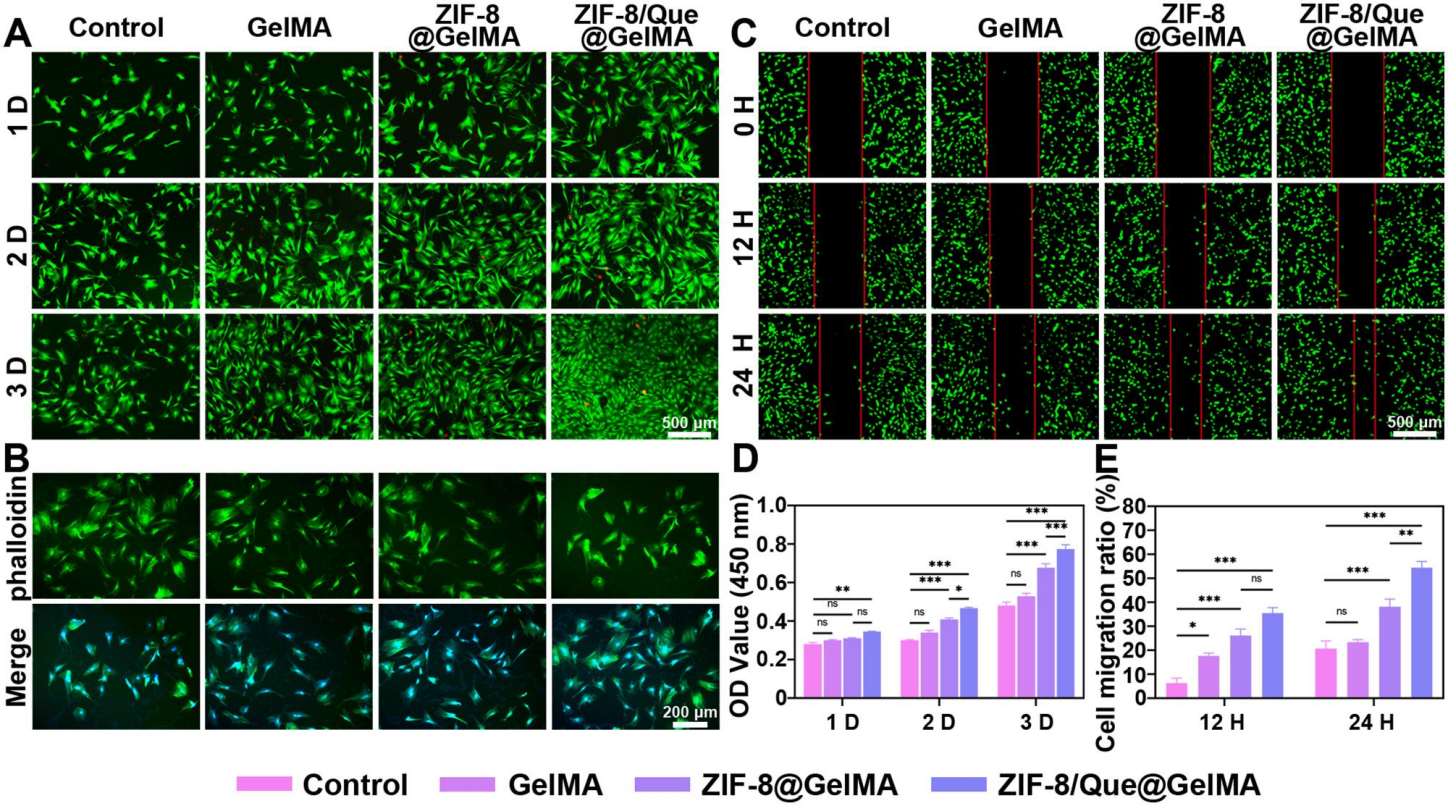
Hydrogel cytocompatibility and immunoregulation *in vitro*. Live/dead staining (**A**) and morphology images (**B**) of BMSCs. (**C**) Scratch experiment images of BMSCs. (**D**) CCK-8 proliferation test of BMSCs. (**E**) Quantitative analysis of scratch experiments.

Cellular biocompatibility is fundamental for osseointegration. We thus investigated the effects of the hydrogel scaffolds on the viability, proliferation, adhesion and migration of BMSCs. Live-dead staining and the CCK-8 kit were used to assess the viability and proliferation of BMSCs on days 1, 2 and 3. Only a few dead cells were observed across all four groups (Figure 4A). Notably, BMSC proliferation was highest in the ZIF-8@GelMA and ZIF-8/Que@GelMA groups, likely owing to the low-concentration Zn^2+^ release. Furthermore, with the release of quercetin, the ZIF-8/Que@GelMA group demonstrated more extraordinary proliferation ability than that of the ZIF-8@GelMA group (Figure 4D).

Compared with observations in the control group, the BMSCs in the ZIF-8/Que@GelMA group extended to form pseudopods based on phalloidin fluorescence staining, resulting in a polygonal appearance and good cell adhesion (Figure 4B). We further conducted a cell scratch assay to simulate the wound healing process *in vitro* (Figure 4C). We observed that the ZIF-8/Que@GelMA group exhibited the most considerable cell migration distance compared to other groups (Figure 4E). This suggests that the coordinated release of Zn^2+^ and quercetin effectively addresses the immune microenvironment requirements, thereby promoting cell migration.

### Effects of hierarchical delivery system on immune regulation

**Figure 5.**
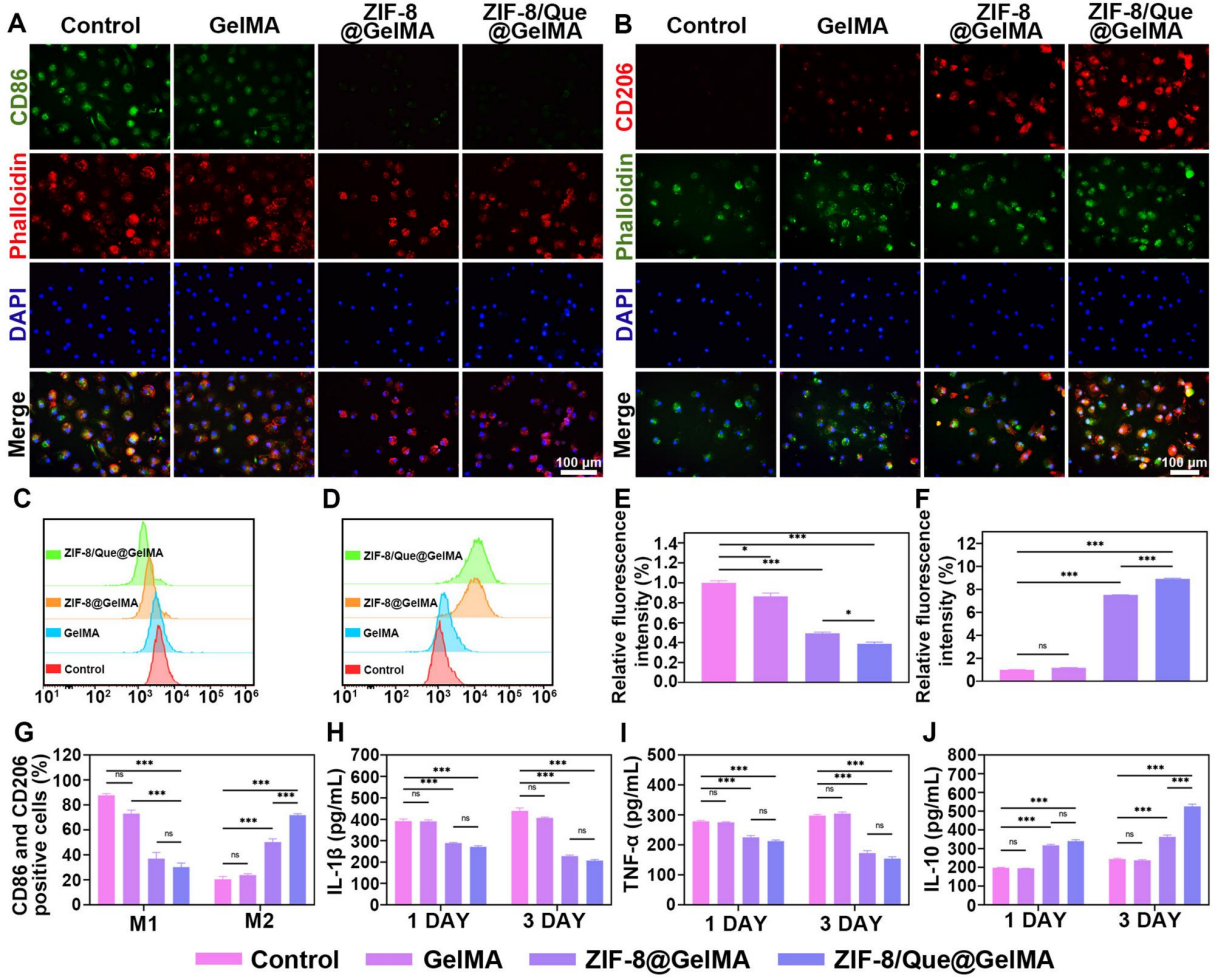
The impact of the external environment on the immune microenvironment. Fluorescence images of M1 (**A**) and M2 (**B**) macrophages. Expressions of CD86 in M1 (**C**) and CD206 in M2 (**D**) by flow cytometry. Quantitative analysis of flow cytometry results for CD86 (**E**) and CD206 (**F**). (**G**) Quantitative analysis of M1 and M2 macrophage polarization. (**H, I, J**) Evaluation of pro-inflammatory factors IL-1β (**H**), TNF-α (**I**), and anti-inflammatory factors IL-10 (**J**) levels in BMSC cells at 1 and 3 days (*n* = 3, *P <* *0.05, **P <* *0.01, and ***P <* *0.001, ns: not significant).

Macrophage polarization is a crucial target for bone immune regulation and serves as a link between inflammatory factors and bone tissue reconstruction [38]. We cultured macrophages in the hydrogel for 3 days and observed their transformation from M0 to M1 and M2 phenotypes by performing immunofluorescence staining. As shown in Figure 5A and Supplementary Figure S2A, LPS-treated macrophages exhibited robust green fluorescence after CD86 (M1 marker) staining in the control group, indicating the activation of M1 macrophages. In contrast, the ZIF-8/Que@GelMA group demonstrated significantly enhanced red fluorescence after CD206 staining (M2 marker), underscoring the superior capacity of ZIF-8/Que@GelMA to induce macrophage polarization toward the M2 phenotype (Figure 5B and Supplementary Figure S2B). However, ZIF-8@GelMA yielded markedly different results. Specifically, its ability to inhibit M1 activation was comparable to that of ZIF-8/Que@GelMA; however, the capacity for M2 activation was diminished (Figure 5G). In addition, flow cytometry also assessed the polarization of M0 macrophages (Figure 5C and D). The ZIF-8/Que@GelMA group exhibited a significantly higher number of M2 macrophages (CD206) compared with those of other groups, followed by that of the ZIF-8/Que@GelMA group (Figure 5E and F). These results indicate that the synergistic anti-inflammatory effects of quercetin and Zn^2+^ are primarily manifested through enhanced M2 polarization.

To further investigate the effects of the composite hydrogel on inflammatory factors, we used LPS stimulation to simulate the bone immune microenvironment. We used ELISA kits to measure IL-1β, TNF-α and IL-10 levels in the supernatant at 1 and 3 days (Figure 5H–J). At both time points, the expression levels of IL-1β and TNF-α were reduced in the ZIF-8/Que@GelMA and ZIF-8@GelMA groups compared with those in the control group, whereas the expression level of IL-10 was elevated. Compared with that in the ZIF-8@GelMA group, there was no significant difference in the anti-inflammatory effect of ZIF-8/Que@GelMA at 1 day. However, considerable enhancement was noted after 3 days. This indicates that ZIF-8/Que@GelMA utilizes low concentrations of Zn^2+^ to exert its anti-inflammatory effects during the early stages of inflammation, and the efficacy of this effect increases rapidly upon quercetin addition.

### *In vitro* immune osteogenesis of the hierarchical delivery system

**Figure 6.**
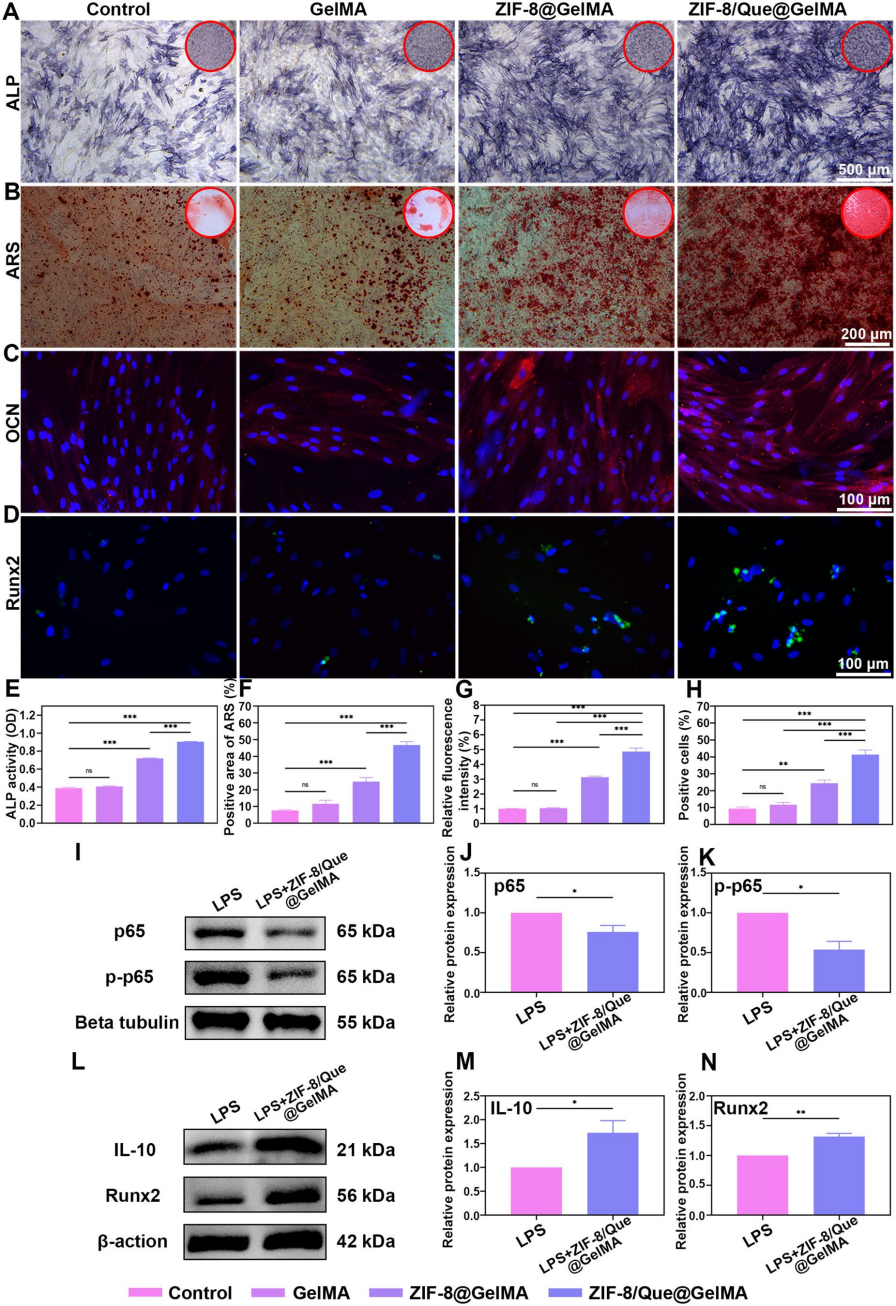
Immunogenic osteogenic differentiation mechanism of hydrogel *in vitro*. (**A**) ALP activity of BMSCs on day 7. (**B**) ARS staining of BMSCs on day 21. Fluorescence images of OCN (**C**) and Runx2 (**D**). Quantitative analysis of ALP (**E**) and ARS (**F**) activity. Quantitative analysis of OCN (**G**) and Runx2 (**H**). (**I**) Protein expression level of p65 and p-p65. Quantitative analysis expression level of p65 (**J**) and p-p65 (**K**). (**L**) Protein expression level of IL-10 and Runx2. The quantitative analysis expression level of IL-10 (**M**) and Runx2 (**N**) (*n* = 3, *P <* *0.05, **P <* *0.01 and ***P <* *0.001, ns: not significant).

ALP is an early osteogenic differentiation marker that provides essential phosphate ions for calcium salt deposition. ALP staining (Figure 6A) and the corresponding quantitative results (Figure 6E) indicated that ZIF-8/Que@GelMA resulted in the most substantial mineralization and bone formation among the four groups. Alizarin Red Staining (ARS) was further employed to assess the content of mineralized bone visually, and the results aligned with those of the ALP activity (Figure 6B and F), thereby validating the osteogenic effect of ZIF-8/Que@GelMA. Additionally, immunofluorescence staining was performed to measure the expression levels of the early osteoblast marker Runx2 and late osteoblast marker OCN on days 7 and 14, respectively (Figure 6C and D). The fluorescence intensities of OCN and Runx2 were most significant in the ZIF-8@GelMA and ZIF-8/Que@GelMA groups (Figure 6G and H), attributed to the sustained release of Zn^2+^. Notably, the fluorescence intensity in the ZIF-8/Que@GelMA group was markedly higher than that in the ZIF-8@GelMA group, ascribed to the synergistic effects of Zn^2+^ and quercetin.

To better understand the molecular mechanisms by which the hierarchical delivery system affects the bone immune microenvironment, we examined the protein expression of the NF-κB signaling pathway using western blotting (Supplementary Figure S3). The results (Figure 6I) and quantitative analysis (Figure 6J and K) show that the expression levels of p65 and p-p65 in the LPS+ZIF-8/Que@GelMA group are lower than those in the LPS group, indicating that the synergistic effect of quercetin and Zn^2+^ inhibits the nuclear translocation of the NF-κB active subunit p65, preventing it from activating the NF-κB signaling pathway by regulating target genes. Inhibiting the NF-κB signaling pathway leads to increased levels of the anti-inflammatory cytokine IL-10 and the essential osteogenic gene Runx2 (Figure 6L–N). This suggests that quercetin and Zn^2+^ can effectively regulate the inflammatory microenvironment, which in turn supports the osteogenic differentiation of BMSCs.

### *In vivo* osteogenic performance assessment

#### In vivo imaging

**Figure 7.**
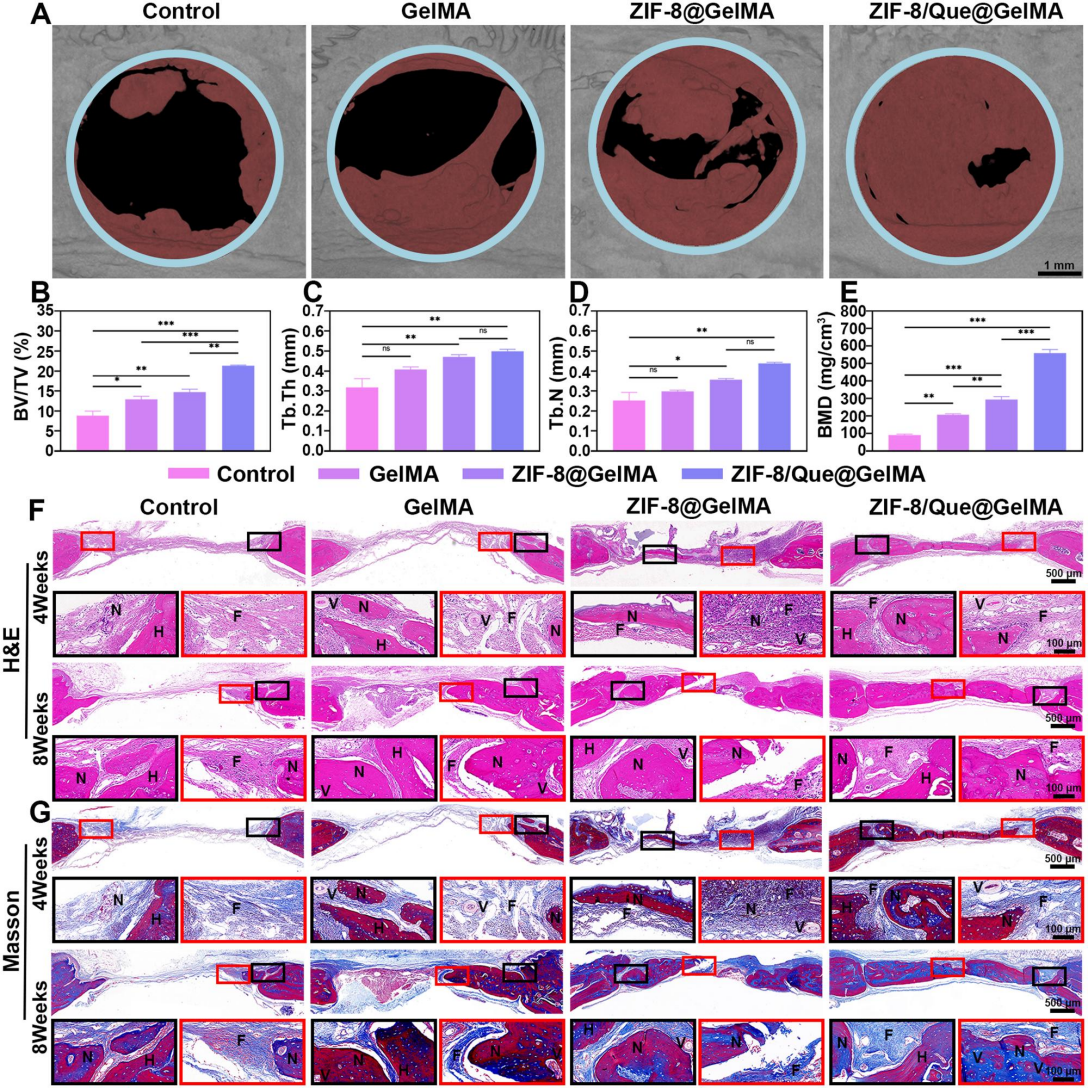
Micro-CT analysis and histological osteogenesis assessment *in vivo*. (**A**) Representative micro-CT 3D reconstructed image of a skull defect. BV/TV (**B**), Tb. Th (**C**), Tb. N (**D**) and BMD (**E**) in the defect area. H&E staining (**F**) and Masson’s staining (**G**). H, N, F and V represent host bone, new bone, fibrous tissue and vessels (*n* = 3, *P <* *0.05, **P <* *0.01 and ***P <* *0.001, ns: not significant).

An electric drill created two full-thickness critical-size bone defects, measuring 5.0 mm, in the rat skull. The plasticity of the composite hydrogel facilitated the injection and filling of the bone defects, which was followed by gelation induced by UV radiation (Supplementary Figure S4). The 3D reconstruction of micro-CT images taken at 8 weeks revealed varying degrees of new bone formation across the groups compared with the initial size, indicated by the blue circle. Notably, the ZIF-8/Que@GelMA group exhibited the largest area of new bone formation within the skull defects (Figure 7A). By contrast, the control group, which was not subjected to hydrogel implantation, displayed only a minimal amount of new bone formation at the edges of the defect, and connectivity was not achieved. The quantitative morphological analysis indicated that ZIF-8@GelMA and ZIF-8/Que@GelMA resulted in the highest BV/TV ratios (Figure 7B), with the new bone tissue exhibiting multidimensional growth patterns. Additionally, similar trends were noted for the Tb. Th, Tb. N, and BMD (Figure 7C–E). Notably, ZIF-8/Que@GelMA had more pronounced advantages over ZIF-8@GelMA regarding the BV/TV and BMD. ZIF-8/Que@GelMA promoted increased bone mass and facilitated the reconstruction of a high-quality, well-organized trabecular bone structure, contributing to enhanced structural stability and load-bearing capacity at the damaged site.

#### In vivo histological evaluation

H&E and Masson staining further corroborated the micro-CT findings. H&E staining (Figure 7F) indicated only a minimal amount of new bone formation along the edge of the host bone at 4 weeks in the control group. In contrast, the other three groups exhibited an increase in both the width and the thickness of the bone defects owing to the hydrogel implantation. Notably, after 4 weeks, the new bone in the ZIF-8/Que@GelMA group effectively covered the defect and continued to grow. Masson staining revealed a similar trend, with most of the bone achieving full-thickness healing after 8 weeks (Figure 7G). Although new bone was observed in the control group at 8 weeks, the quantity of new collagen fibers was decreased compared with that at 4 weeks, indicating that self-repair was not feasible. The GelMA group demonstrated less new bone formation than did the ZIF-8@GelMA and ZIF-8/Que@GelMA groups. It exhibited the substantial presence of collagen fibers within the defect, which was attributed to the robust support provided by the hydrogel scaffold for osteoblast activity. In the bone tissue defects of the ZIF-8@GelMA and ZIF-8/Que@GelMA groups, numerous dense, blue-stained mature bones and red-stained bone-like islands were evident, which were particularly pronounced in the ZIF-8/Que@GelMA group. At 8 weeks, large lamellar bones with abundant vascular infiltration, resembling natural bone tissue, were observed in the ZIF-8/Que@GelMA group.

#### In vivo osteogenic gene expression

**Figure 8.**
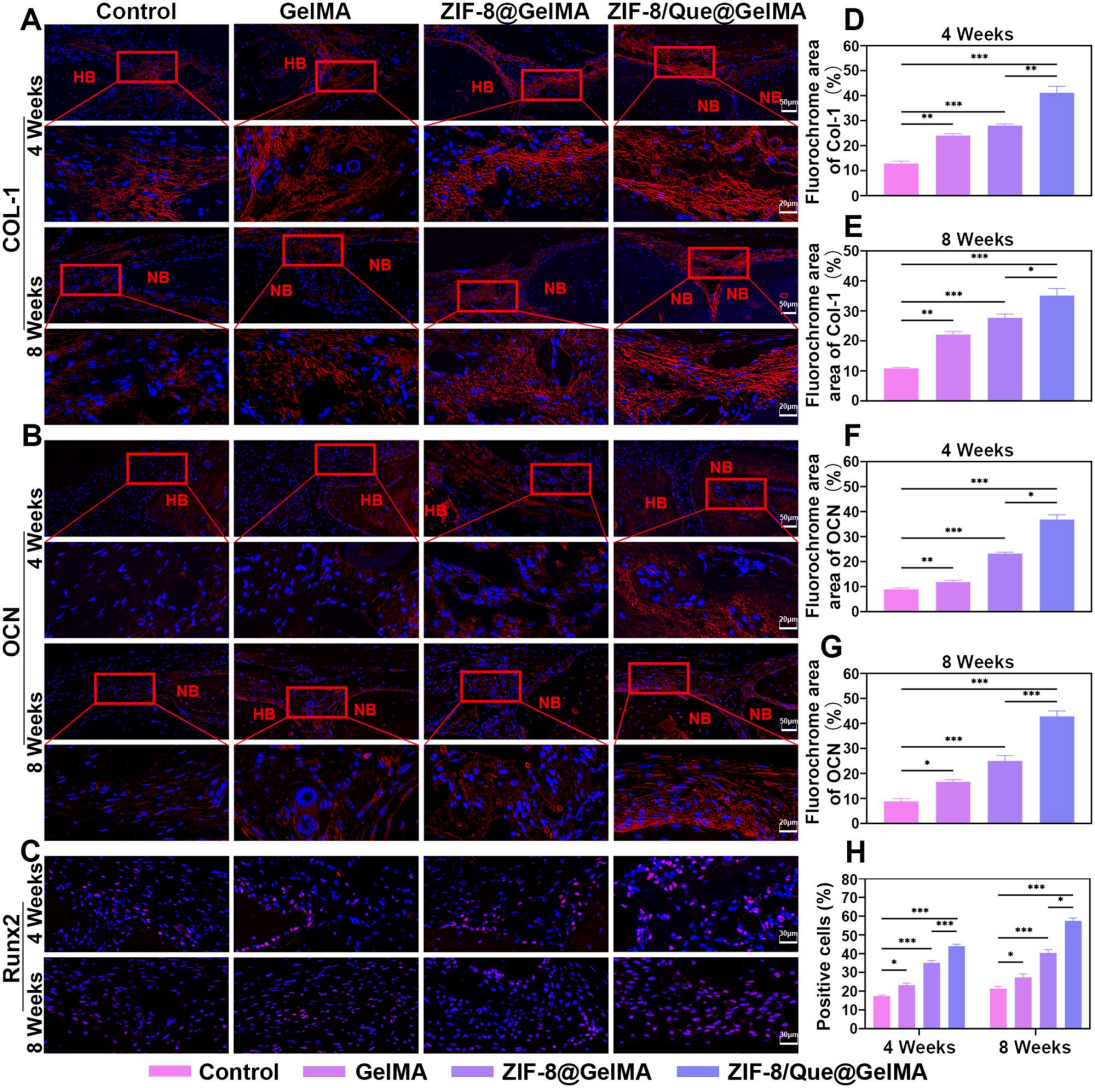
Immunofluorescence staining of osteogenic ability at 4 and 8 weeks. Fluorescence images of osteogenic differentiation markers COL-1 (**A**) and OCN (**B**). HB and NB represent host bone and new bone, respectively. (**C**) Fluorescence images of the intranuclear osteogenic differentiation marker Runx2. Quantitative analysis of COL-1 (**D, E**), OCN (**F, G**) and Runx2 (**H**) at 4 and 8 weeks (*n* = 3, *P <* *0.05, **P <* *0.01 and ***P <* *0.001, ns: not significant).

At 4 and 8 weeks after hydrogel implantation, we conducted immunofluorescence staining to examine the expression of the osteogenic differentiation markers COL-1 and OCN within and outside the osteoblast cytoplasm, as well as Runx2 in the nucleus (Figure 8A–C). This approach allowed us to assess whether bone repair was progressing normally and to determine the speed and quality of new bone formation. The overall expression levels of COL-1 and Runx2, as early indicators of osteogenesis, in each group exhibited a downward trend from 4 to 8 weeks, suggesting that bone repair was entering a relatively mature phase (Figure 8D, E and H). Conversely, the overall expression level of the late osteogenesis marker OCN increased from 4 to 8 weeks, except in the control group, which showed a plateau in osteogenic differentiation (Figure 8F and G). Notably, the fluorescence area, after staining with the three osteogenic differentiation markers, was significantly increased in the ZIF-8/Que@GelMA group compared with that in the other groups, correlating with the imaging and histological findings. These results demonstrate that ZIF-8/Que@GelMA promotes new bone reconstruction and helps maintain bone density and strength.

### *In vivo* bone immune regulation

**Figure 9.**
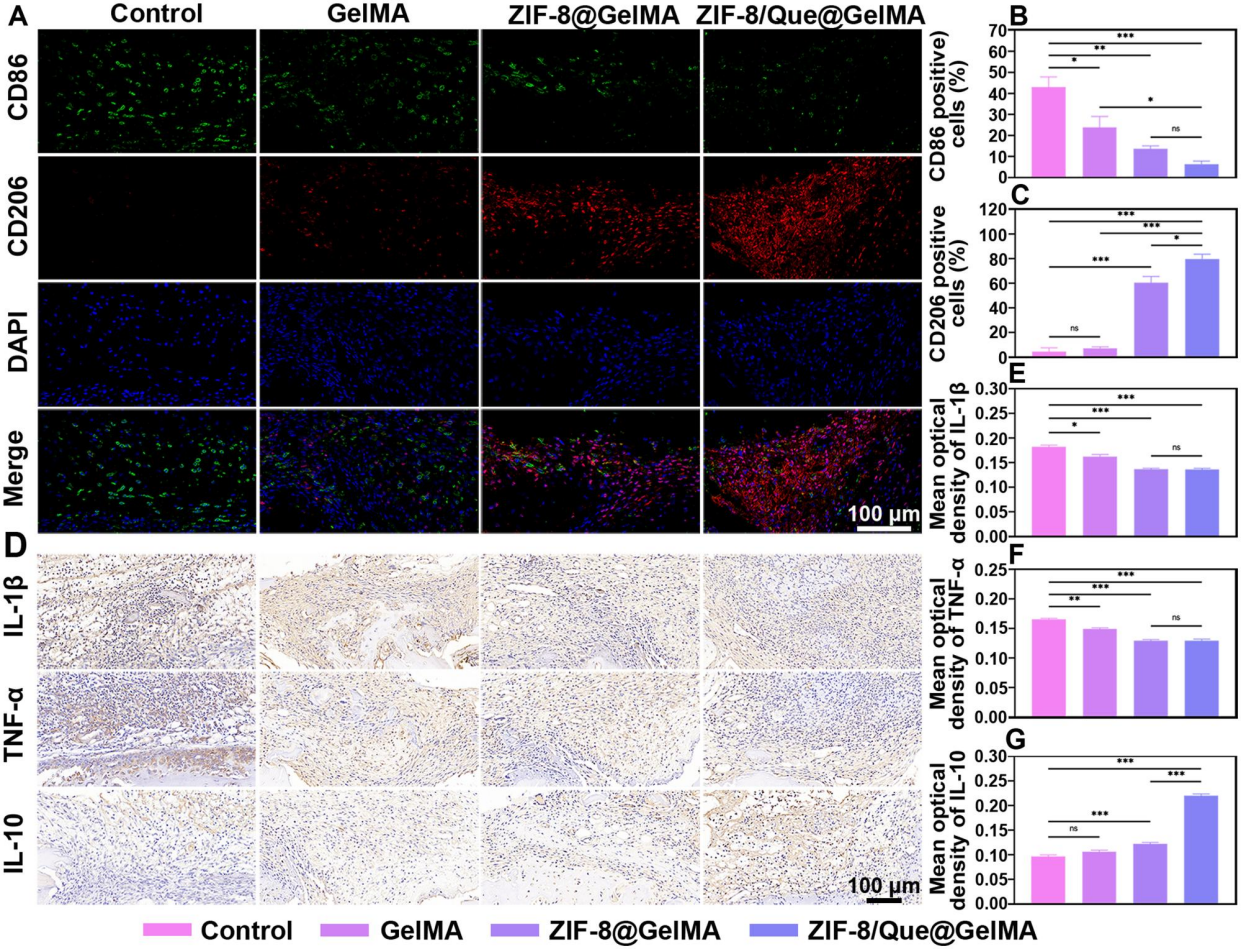
Evaluation of the immunoregulatory effect. (**A**) Fluorescence images of M1 and M2 macrophages at day 7. (**B** and **C**) Quantitative analysis of M1 and M2 macrophage polarization. (**D**) Immunohistochemical staining of the IL-1β, TNF-α and IL-10 with dark brown areas at day 7. (**E**–**G**) Quantitative analysis of IL-1β, TNF-α and IL-10. (*n* = 3, *P <* *0.05, **P <* *0.01 and ***P <* *0.001, ns: not significant).

Macrophage polarization in the bone defect was also assessed after 7 days via immunofluorescence staining (Figure 9A). The control group had the most pro-inflammatory M1 cells (CD86) and the fewest anti-inflammatory M2 cells (CD206). Compared with the control group, the GelMA group exhibited a reduction in M1 cells, although no significant difference in M2 cell levels was observed. Moreover, both ZIF-8@GelMA and ZIF-8/Que@GelMA significantly reduced the number of M1 cells, with no notable difference between the two treatment groups (Figure 9B). Further, the ZIF-8/Que@GelMA group had substantially more M2-positive cells than the other groups (Figure 9C). These results indicate that the hydrogel scaffold might serve as a physical barrier to mitigate inflammation *in vivo* and that M2 polarization was markedly enhanced following the incorporation of Zn^2+^ and quercetin.

Upon activation, macrophages release numerous inflammatory factors. We thus conducted immunohistochemical staining for the inflammatory factors IL-1β, TNF-α and IL-10 at 7 days (Figure 9D). Similar to the macrophage polarization trends, the staining intensity of the pro-inflammatory factors IL-1β and TNF-α was higher in the control group. Notably, the positive IL-1β and TNF-α staining in the ZIF-8@GelMA and ZIF-8/Que@GelMA groups was significantly reduced owing to the active involvement of Zn^2+^ in the inflammatory process (Figure 9E and F). Moreover, adding quercetin resulted in the most intense IL-10-positive staining in the ZIF-8/Que@GelMA group (Figure 9G). These findings suggest that Zn^2+^ exerts a strong inhibitory effect on pro-inflammatory factors through its sustained release from the hydrogel. Furthermore, quercetin and Zn^2+^ function synergistically to promote the production of many anti-inflammatory factors, thus playing a decisive role in the anti-inflammatory response. H&E staining was also performed on the primary organs of the rats 8 weeks after model establishment. No significant pathological changes or inflammatory reactions were observed in the heart, liver, spleen, lungs or kidneys (Supplementary Figure S5). These observations suggest that ZIF-8/Que@ZIF-8 exhibits good biocompatibility and is not toxic following implantation.

## Discussion

In bone tissue engineering, biomaterial design has evolved from primarily focusing on biocompatibility to incorporating immunomodulatory functions. In this study, we prepared nanoparticles containing the anti-inflammatory materials ZIF-8 and quercetin, which were subsequently embedded in a GelMA hydrogel. This combination resulted in anti-inflammatory effects in distinct temporal and spatial contexts, fostering a favorable inflammatory microenvironment. Both *in vitro* and *in vivo* experiments demonstrated that the ZIF-8/Que@GelMA hierarchical delivery system facilitates the controllable transformation of the immune microenvironment and accelerates bone regeneration in defects.

Hybrid scaffolds serve as the foundation for achieving immune regulation in tissue engineering. By integrating hydrogels with anti-inflammatory materials or drugs [23, 39], these scaffolds enable the continuous and directional release of anti-inflammatory substances, facilitating the accumulation of immune cells and enhancing the activation and proliferation of osteoblasts [28, 40]. Notably, the GelMA hydrogel itself did not produce anti-inflammatory factors. Interestingly, our *in vivo* experiments demonstrated that GelMA inhibited M1 polarization and reduced the concentration of pro-inflammatory factors without affecting anti-inflammatory factor production. This finding aligns with Donaldson et al. [41], indicating that GelMA hydrogels can create a physical barrier between damaged tissues and the external environment, thereby preventing further exacerbation of the inflammatory process. However, despite the adhesiveness and 3D structure of the hydrogel, which promotes effective interactions with the surrounding biological tissues and facilitates cell attachment and growth [6, 31], this cannot compensate for the osteogenic capacity deficit caused by its limited anti-inflammatory effect. Incorporating ZIF-8/Que nanoparticles into the hydrogel resulted in a more granular surface and cross-section and an increased pore size. While maintaining the favorable elasticity of GelMA, this modification allowed the hydrogel to withstand pressure several times, thereby providing a stable environment conducive to cell growth. These findings indicate that ZIF-8/Que@GelMA can effectively fill tiny gaps and pores, adhering firmly to the surface of the bone tissue and enabling the efficient loading and directional release of anti-inflammatory drugs to the inflammatory site.

To construct an optimal model of bone immune regulation to achieve bone regeneration, it is essential to establish a low anti-inflammatory microenvironment that activates repair mechanisms and an anti-inflammatory microenvironment that promotes osteoblast differentiation. Appropriate levels of pro-inflammatory factors introduced by an early low-anti-inflammatory environment are critical for maintaining the balance between bone and immune responses [20]. Following bone defect occurrence, local immune cells, such as macrophages, rapidly accumulate at the defect site. These immune cells secrete pro-inflammatory factors (IL-1, TNF-α) to recruit stem cells and reparative cells to the damaged area [42]. However, most current bone immune biomaterials utilize the sustained-release properties of carriers to deliver metal ions or anti-inflammatory drugs continuously. This approach maintains anti-inflammatory concentrations at elevated levels, which could inhibit the ability of the body to fully activate a pro-inflammatory response during the early stages, thereby potentially limiting its positive effects [38]. ZIF-8 primarily encapsulates the loaded drug through physical embedding rather than covalent bonding, trapping the drug within its porous framework. The stability of the ZIF-8 framework depends on the bonds formed between Zn^2+^ and imidazole ligands [43]. Under acidic conditions, the protonation of imidazole ligands disrupts these bonds, causing the release of Zn^2+^. As more Zn^2+^ is released, it leads to structural collapse and the release of the encapsulated drug [44]. This mechanism is fundamental to the sequential release of drugs and their responsiveness to environmental changes. In this study, the ZIF-8/Que@GelMA maintained a low concentration of Zn^2+^ during the early stages of inflammation. Zn^2+^ has been shown to inhibit the secretion of TNF-α and IL-1β in M1 macrophages while promoting the expression of transforming growth factor-β (TGF-β) in M2 macrophages [45, 46]. Relevant *in vitro* studies indicate that the effective concentration of Zn^2+^ typically ranges from 1 to 50 µM (0.06–3.26 µg/mL), which can significantly suppress the inflammatory response [42]. However, at 1.39 μg/mL, macrophages in the initial stage (1–3 days) tend to polarize toward the M1 phenotype [47]. Moreover, at 1.59 μg/mL, Zn^2+^ can induce significant cytotoxic effects [48], and concentrations exceeding 100 μM (6.54 μg/mL) can lead to cell death [49]. The Zn^2+^ concentration was maintained below 1.0 µg/mL using a dual sustained-release mechanism. This approach ensured the safety associated with the pH-responsive release of Zn^2+^ from ZIF-8 and maximized the beneficial immune balance associated with low anti-inflammatory effects during the early stage. To further optimize early immune regulation, another study employed a hydrogel coating as a “dam” to intercept the release of IL-4 via electrospinning 3 days before bone defect creation, thereby sustaining a moderate inflammatory response through low anti-inflammatory effects in the early stages of inflammation [50]. The ZIF-8/Que@GelMA hierarchical release system intelligently regulated Zn^2+^ release, effectively initiating immune regulation conducive to bone defect repair. This process would promote the interaction between pro-inflammatory factors and the proliferation of BMSCs to prepare for subsequent bone tissue repair adequately.

The polarization of macrophages is crucial for bone regeneration mediated by the immune system. Previous studies have demonstrated that quercetin promotes macrophage polarization to the M2 type by activating pattern recognition receptors like TLR2 [17, 51]. We focus on how quercetin affects the inflammatory microenvironment and its immunomodulatory effects. For instance, quercetin inhibits the Mincle/Syk/NF-κB signaling pathway, which reduces LPS-induced inflammatory responses in macrophages [52]. The NF-κB signaling pathway plays a central role in the inflammatory response, as it can regulate the expression of various inflammatory factors. Quercetin primarily inhibits the NF-κB signaling pathway by interfering with the phosphorylation and degradation of IκB proteins. This inhibition prevents NF-κB dimers from being released from their cytoplasmic complex with IκB. As a result, NF-κB cannot enter the nucleus to exert its transcriptional regulatory effects [53]. Quercetin also directly inhibits the phosphorylation of p65, further reducing NF-κB’s transcriptional activity because phosphorylated p65 can bind DNA more effectively and activate gene transcription [54]. In our study, ZIF-8/Que effectively reduced the overall activity of the NF-κB signaling pathway by inhibiting the phosphorylation of p65. This inhibition led to an increase in anti-inflammatory factors, such as IL-10, which promote M2 macrophage polarization. The polarized M2 macrophages continue to secrete IL-10, thereby establishing a positive feedback mechanism that enables them to play a crucial role in maintaining the local immunosuppressive microenvironment [55].

Zn^2+^ plays a crucial role in regulating inflammation. It not only inhibits the activation of Th17 cells via the IL-6/STAT3 signaling pathway and modulates the expression of pro-inflammatory cytokines such as IL-17 [56] but also influences the immune response of cells by activating the PI3K/AKT and MAPK signaling pathways, thereby regulating the activity of downstream signaling molecules [57, 58]. Similarly, Zn^2+^ has been demonstrated to inhibit macrophage M1 polarization by reducing NF-κB activation and its target genes, including TNF-α and IL-1β, highlighting their strong inhibitory effect on pro-inflammatory factors [59]. However, adding quercetin did not significantly improve ZIF-8’s inhibitory effect on pro-inflammatory factors [24]. Our study supports this observation, suggesting that the potent immunomodulatory ability of ZIF-8@Que mainly comes from the anti-inflammatory factors released by quercetin, which aids in reprogramming macrophages [60]. However, the synergistic effect of Zn^2+^ and quercetin should not be underestimated. Zn^2+^ can enhance the intracellular uptake of quercetin, thereby improving its bioavailability [61]. Due to its structural characteristics, quercetin can form complexes with various metal ions, demonstrating particularly effective chelating ability at the 3-hydroxy and 4-carbonyl positions. The formation of these complexes can significantly enhance the utilization potential of quercetin [62]. Moreover, the degradation of ZIF-8 in physiological environments plays a crucial role in the mechanism underlying its effects on bone formation. Soaking ZIF-8 in simulated body fluids induces the formation of large crystals, and the rapid growth of these crystals provides additional biomineralization sites, thereby promoting bone formation [63]. Our study also revealed the excellent mineralization-inducing and osteogenic capabilities of ZIF-8@GelMA and ZIF-8/Que@GelMA. Notably, although the hydrogel scaffold facilitated osteoblasts’ network-like adhesion and migration, trabeculae exhibiting osteogenic morphology did not show any advantages after quercetin incorporation. However, the significant improvements observed in the BV/TV ratio and BMD after the ZIF-8/Que@GelMA application suggest that quercetin has relatively good osteogenic capabilities. Zhang et al. found that quercetin promotes the proliferation and osteogenic differentiation of BMSCs and that its osteogenic effect is partially regulated by the miR-206/Cx43 pathway [64]. We propose that the potent immunomodulatory effect of quercetin is pivotal for osteogenesis, which aligns with the findings of most current studies [7, 16].

In addition, we conducted fluorescence staining to investigate the osteogenic mechanisms following quercetin loading. The ZIF-8/Que@GelMA system resulted in a consistent upward trend in the expression of several osteogenic genes. These findings align with previous studies indicating that quercetin protects osteoblasts by reducing intracellular oxidative stress levels and releasing inflammatory factors, thereby enhancing the expression of Runx2, which promotes bone growth [65]. As a master regulator, Runx2 facilitates the expression of COL-1 and OCN, which are essential for bone development [66]. Runx2 can enhance the transcriptional accessibility of the promoter regions of *COL-1* and *OCN* by altering the chromatin structure [67]. The superior bone immune regulatory effects exerted by ZIF-8/Que@GelMA resulted in the highest expression of Runx2 in the nuclei of osteoblasts surrounding mature bones during the early stages of osteogenesis (4 weeks), consequently upregulating COL-1 expression and promoting its synthesis. This process establishes a foundation for subsequent bone mineralization and OCN expression (8 weeks). Notably, as osteoblasts differentiate and mature, the expression level of Runx2 gradually decreases to facilitate the normal development and functional maintenance of bone tissue [68].

It is essential to consider bone immune regulatory mechanisms during bone repair. In line with this, we observed significant therapeutic effects. The bone immune response constitutes a complex network that involves multiple cell types and signaling pathways. However, our current understanding of these critical connections is preliminary. Therefore, future research should be more comprehensive and systematically explore how this new system regulates interactions between immune cells and its influence on related signaling pathways. Furthermore, although the animal experimental model used in this study partially simulated the pathophysiological processes associated with bone defects, it still differs from actual clinical scenarios. Consequently, in follow-up studies, larger animal models that are more clinically relevant must be established to assess this treatment's safety and efficacy accurately.

In summary, ZIF-8 functions as a carrier and initial layer for the sustained release of anti-inflammatory metals. Low-concentration sustained release maintains a mild anti-inflammatory effect that facilitates initial immune regulation. Subsequently, in response to changes in the inflammatory environment, the second layer of quercetin within ZIF-8 is released to exert a potent synergistic anti-inflammatory effect. This hierarchical delivery system establishes an optimal environment for maintaining the bone–immune balance and promoting bone regeneration.

## Conclusion

An injectable nanocomposite hydrogel system was developed and characterized to modulate the osteoimmune microenvironment. For this system, a GelMA hydrogel was initially utilized to facilitate the slow release of Zn^2+^ from the shell of ZIF-8 nanoparticles, achieving a low anti-inflammatory effect optimal for the body during the early stages of inflammation. Subsequently, quercetin is released from ZIF-8 in response to changes in the microenvironment, synergizing with Zn^2+^ to regulate anti-inflammatory responses, maintain the bone immune balance and promote the repair of bone defects. Moreover, the performance of this nanocomposite hydrogel system was thoroughly investigated through a series of experiments that demonstrated its excellent biocompatibility, controllable release characteristics and significant efficacy in bone defect repair. This nanocomposite hydrogel system is the first to employ biomaterials for sequential microenvironment regulation, thereby providing a novel foundation for treating bone defects and demonstrating considerable potential for clinical applications.

## Supplementary Material

rbaf006_Supplementary_Data
