# Supplementary material for: Spatiotemporal regulation of the bone immune microenvironment via a ‘Zn2+-quercetin’ hierarchical delivery system for bone regeneration
Source: Regen Biomater. 2025 Feb 13;12:rbaf006. doi: 10.1093/rb/rbaf006 (PMC11925500; doi:10.1093/rb/rbaf006)
Supplement: rbaf006_Supplementary_Data [file rbaf006_supplementary_data.docx]

**Supplementary Material:**

# Spatiotemporal Regulation of the Bone Immune Microenvironment via a ‘Zn2+–quercetin’ Hierarchical Delivery System for Bone Regeneration

Hengliang Sun^1,2,7^**^†^**, Yedan Chen^2,3^**^†^**, Xiaoqin Sang^3,6^, Qingxiang Liu^3,6^, Haoran Yu^3^, Shaojun Hu^4,^*, Yingji Mao^2,3,5,^*, Li Zhang^1,2,^*

^1^ Anhui Medical University, Hefei 230032, Anhui, China

^2^ Department of Plastic Surgery, the First Affiliated Hospital of Bengbu Medical University, Bengbu 233004, Anhui, China

^3^ Anhui Nerve Regeneration Technology and Medical New Materials Engineering Research Center, School of Life Sciences, Bengbu Medical University, Bengbu 233030, Anhui, China

^4^ Huaiyuan County People's Hospital, Bengbu, Anhui 233400, China

^5^ Anhui Provincial Key Laboratory of Tumor Evolution and Intelligent Diagnosis and Treatment, Bengbu Medical University, Bengbu 233030, Anhui, China

^6^ Department of Rehabilitation Medicine, the First Affiliated Hospital of Bengbu Medical University, Bengbu 233004, Anhui, China

^7^ Second People’s Hospital of Wuhu City, Wuhu 241001, Anhui, China

**^†^** Hengliang Sun and Yedan Chen contributed equally to this work.

***Corresponding Authors:**

Shaojun Hu, Huaiyuan County People's Hospital, Bengbu 233400, Anhui, China; Email: hushao8971881@163.com.

Yingji Mao, Anhui Nerve Regeneration Technology and Medical New Materials Engineering Research Center, School of Life Sciences, Bengbu Medical University, Bengbu 233030, Anhui, China; Email: myj123@bbmu.edu.cn.

Li Zhang, Department of Plastic Surgery, The First Affiliated Hospital of Bengbu Medical College, Bengbu 233004, Anhui, China; Email: drzhangli65@163.com.


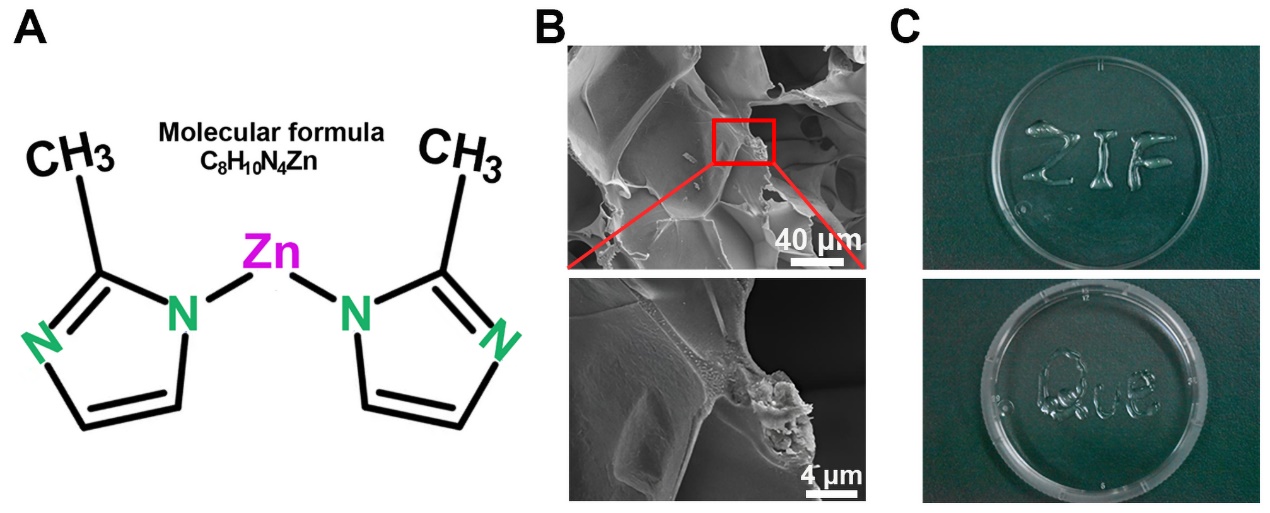


**Figure S1.** (A) The molecular formula of ZIF-8. (B) SEM images of ZIF-8@GelMA hydrogel enlargement. (C) The plasticity of hydrogel materials.


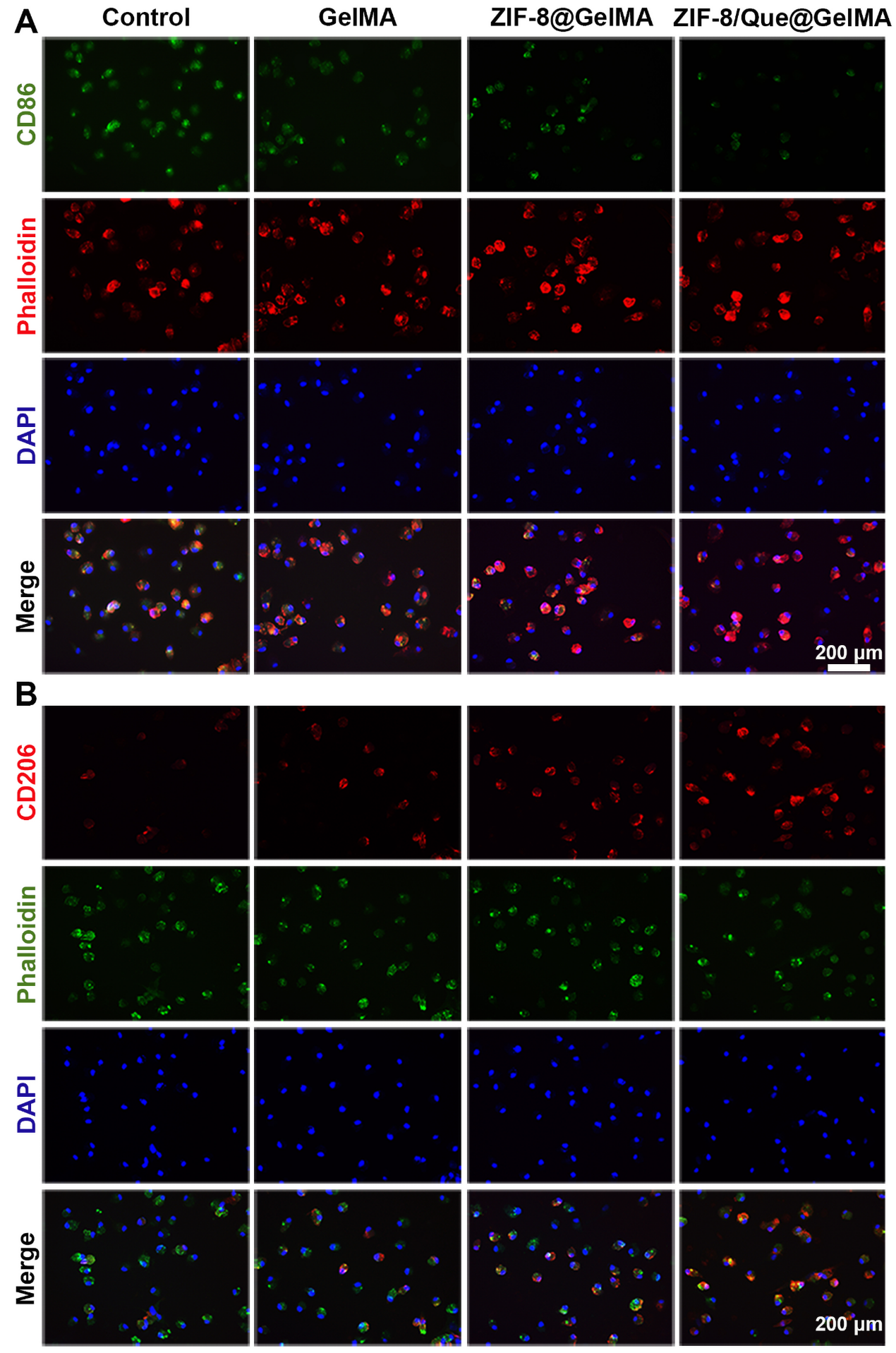


**Figure S2.** 200x fluorescence images of M1 (A) and M2 (B) macrophages.


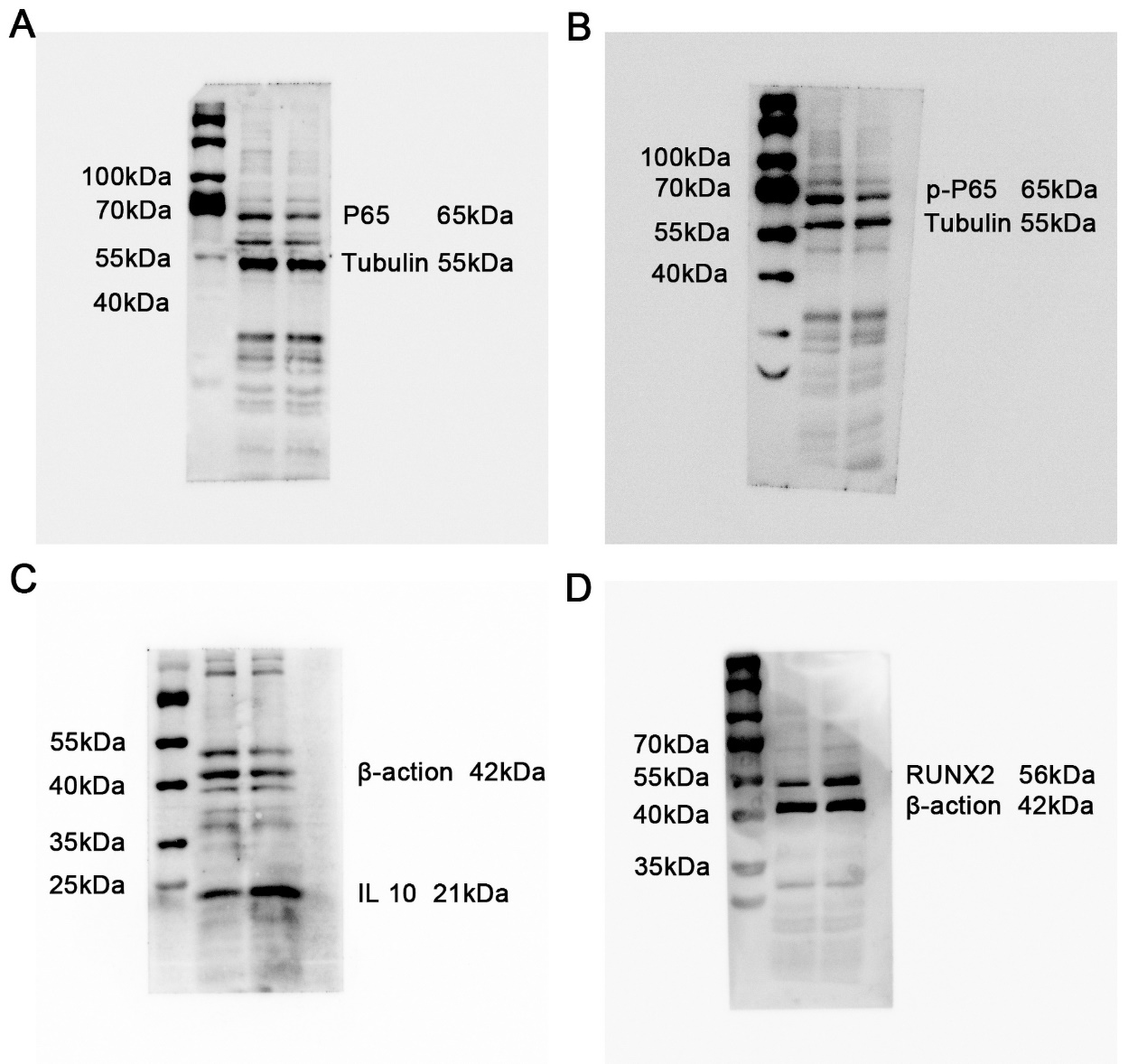


**Figure S3.** Uncropped whole film image of p65 (A), p-p65 (B), IL-10 (C) and Runx2 (D).


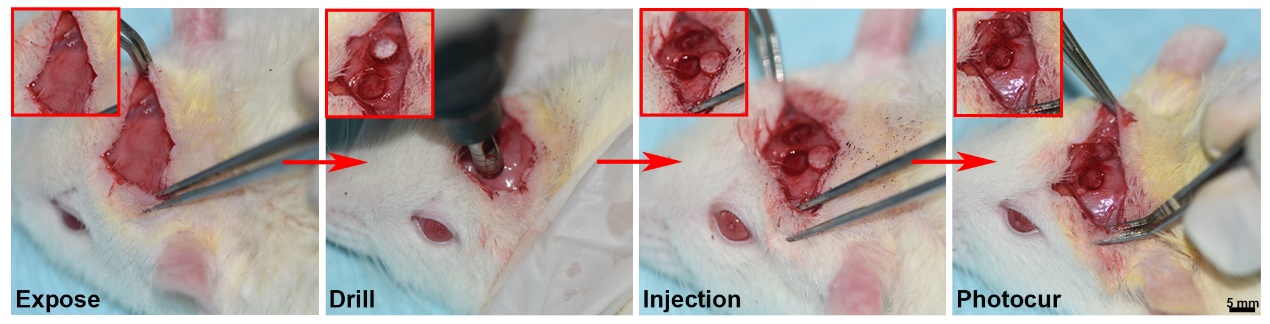


**Figure S4.** Skull defect repair in a rat model.


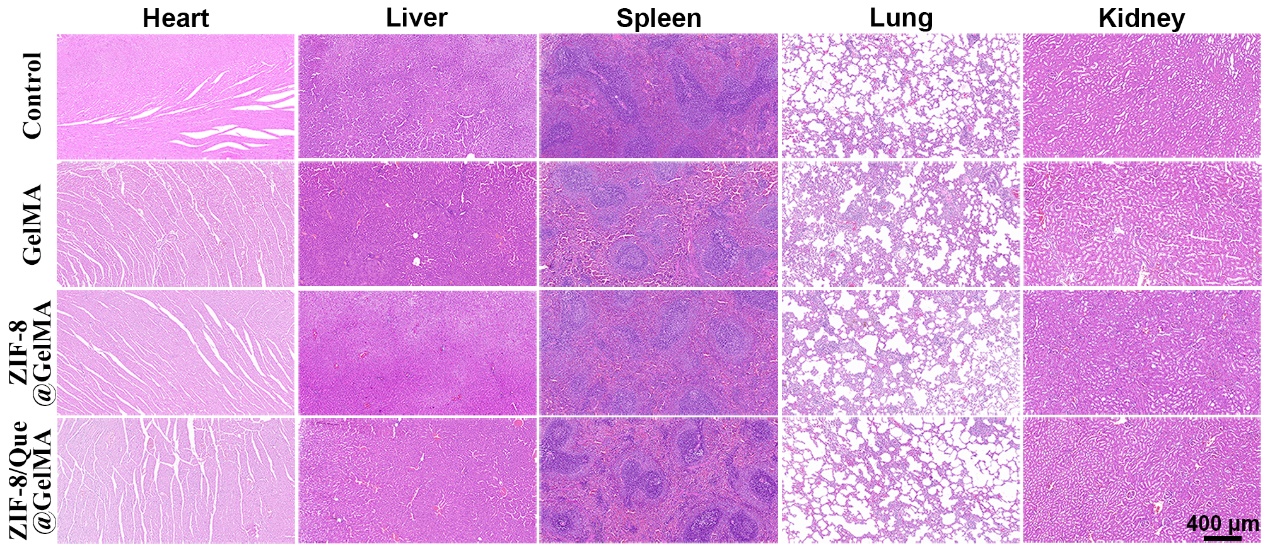


**Figure S5.** H&E staining of major organs (heart, liver, spleen, lung, and kidney) 8 weeks after implantation.
